# Identification, molecular characterization and phylogenetic analysis of a novel nucleorhabdovirus infecting *Paris polyphylla* var. *yunnanensis*

**DOI:** 10.1038/s41598-023-37022-2

**Published:** 2023-06-20

**Authors:** Jingyu Hu, Tianli Miao, Kaijuan Que, Md. Siddiqur Rahman, Lei Zhang, Xian Dong, Pengzhang Ji, Jiahong Dong

**Affiliations:** 1 School of Chinese Materia Medica and Yunnan Key Laboratory of Southern Medicinal Resource, Yunnan University of Chinese Medicine, Yunnan, 650500 China; 2 Institute of Medicinal Plant Cultivation, Academy of Southern Medicine, Yunnan University of Chinese Medicine, Yunnan, 650500 China; 3 Plant Pathology Division, Bangladesh Agricultural Research Institute, Gazipur, 1701 Bangladesh

**Keywords:** Plant molecular biology, Molecular biology, Plant sciences

## Abstract

A novel betanucleorhabdovirus infecting *Paris polyphylla* var. *yunnanensis*, tentatively named Paris yunnanensis rhabdovirus 1 (PyRV1), was recently identified in Yunnan Province, China. The infected plants showed vein clearing and leaf crinkle at early stage of infection, followed by leaf yellowing and necrosis. Enveloped bacilliform particles were observed using electron microscopy. The virus was mechanically transmissible to *Nicotiana bethamiana* and *N. glutinosa*. The complete genome of PyRV1 consists of 13,509 nucleotides, the organization of which was typical of rhabdoviruses, containing six open reading frames encoding proteins N–P–P3–M–G–L on the anti-sense strand, separated by conserved intergenic regions and flanked by complementary 3′-leader and 5′-trailer sequences. The genome of PyRV1 shared highest nucleotide sequence identity (55.1%) with Sonchus yellow net virus (SYNV), and the N, P, P3, M, G, and L proteins showed 56.9%, 37.2%, 38.4%, 41.8%, 56.7%, and 49.4% amino acid sequence identities with respective proteins of SYNV, suggesting RyRV1 belongs to a new species of the genus *Betanucleorhabdovirus*.

## Introduction

*Paris polyphylla* is a perennial herb belonging to the tribe Parideae of the family Melanthiaceae. Rhizome paridis (called “Chonglou” in Chinese) is a precious traditional Chinese medicine as the raw sources of several well-known Chinese medicines such as “Yunnan baiyao”, “Jideshengsheyaopian” and “Gongxuening Capsules”, which are used as haemostatic, antalgic and antipyretic treatment, respectively. *P. polyphylla* contains chemical constituents of steroidal saponins, β-ecdysone, polysaccharide, flavone glycoside and other active compounds^1^. Modern pharmacological studies have revealed that these chemical constituents have antimicrobial, antiviral, antihelminthic and antineoplastic activities^2–6^. *P. polyphylla* var. *yunnanensis* is distributed throughout the Yunnan-Guizhou Plateau, Sichuan province, and Chongqing city. Due to the economic and medicinal value, wild *P. polyphylla* var. *yunnanensis* was reaped excessively. As a result, wild *P. polyphylla* var. *yunnanensis* is very scarce. So, artificial cultivation of the plant is crucial to meet market demand. The large-scale cultivation of *P. polyphylla* var. *yunnanensis* may lead to the emerging of new fungal, bacterial and viral diseases that have the potential to negatively impact the yield and quality. Till to date, six viruses have been reported to infect the *P. polyphylla* var. *yunnanensis* that cause the yield losses. They include Paris polyphylla virus X (PPVX), Paris mosaic necrosis virus (PMNV), pepper mild mottle virus (PMMoV), cowpea aphid-borne mosaic virus (CABMV), Paris virus 1 (ParV1), and Yunnan paris negative-stranded virus (YPNSV)^7–12^.

Viruses of the *Rhabdoviridae* family in the order *Mononegavirales* are important pathogens of animals and plants. The virions are typically bullet-shaped or bacilliform 100–430 nm length and 45–100 nm wide composed of a helical nucleocapsid surrounded by a matrix layer and a lipid envelope^13,14^. Their genomes consist of negative-sense single-strand RNA (− ssRNA) 10.8–16.1 kb in length with partially complementary termini^15^. Taxonomically, plant rhabdoviruses are assigned to the subfamily *Betarhabdovirinae* that includes 6 genera (*Alphanucleorhabdovirus*, *Betanucleorhabdovirus*, *Cytorhabdovirus*, *Dichorhavirus*, *Gammanucleorhabdovirus* and *Varicosavirus*) based on the replication sites and morphogenesis in the cytoplasm or nucleus of infected cells, and their genome architecture^14–16^. Betanucleorhabdovirus genomes encode 6 canonical proteins in the conserved order of 3′-to-5′: nucleoprotein (N), phosphoprotein (P), movement protein (P3), matrix protein (M), glycoprotein (G) and RNA-dependent RNA polymerase (L). The genome lacks additional accessory open reading frames (ORFs). The genes are flanked by conserved junction regions 3′-AUUCUUUUU GG UUG-5′ separating their genes, and complementary 3′-leader and 5′-trailer sequences^15,17,18^. Recently, with the application of High Throughput Sequencing (HTS), more and more plant rhabdoviruses were discovered^19,20^. The previously reported betanucleorhabdoviruses infect dicot and monocot plants and several of them are transmitted by aphids in which they also replicate^21^.

In this study, we used the HTS platforms and RT-PCR to determine the complete genome of a new betanucleorhabdovirus infecting *P. polyphylla* var. yunnanensis, provisionally named Pairs yunnanensis rhabdovirus 1 (PyRV1) based on analysis of genomic organization, sequence homology and phylogeny.

## Methods

### Virus source

A field survey was conducted to find out medicinal plant diseases in Yunnan province, China in 2019. During the survey, virus infected *Paris polyphylla* var. *yunnanensis* plant samples were collected from the medicinal plant plantation in QuJing city of YunNan province. The diseased leaf material was homogenized at ratio 1:5 (w/v) in 0.1 mol/L sodium phosphate Buffer, pH7.0, with carborundum powder as an abrasive agent. Then it was mechanically smeared with the pestle onto the young leaves of *Nicotiana benthamiana*, *N. glutinosa*, *N. tabacum* var. K326 and *Sonchus oleraceus* and injected with a medicinal injector into the leaf vein of *P. polyphylla var. yunnanensis* to determine if the virus was sap-transmitted.

Experimental research on plant samples, including the supply of plant material, complies with institutional, national and international guidelines and legislation.

### Negative stain and electron microscopy observation

For electron microscopy, 0.1 g symptomatic leaves from *P. polyphylla* var. *yunnanensis* were directly cut in 20 µL 2.5% isoamyl alcohol until the tissues was completely homogenized. A pioloform carbon-coated copper grid was floated for 3.5 min on the crude sap to absorb the viral particles. The grids were then transferred onto one drop of 20 µL negative staining Buffer (2% ammonium molybdate at pH 6.5) and stained for 2 min. Finally, the grids were dried by absorbing moisture with filter paper. The virions were examined under a transmission electron microscope (FEI TECNAI G2, ThermoFisher Scientific, Hillsboro, Oregon, USA).

### High throughput sequencing and read assembly

Total RNA was extracted from the diseased *P. polyphylla* var. *yunnanensis* leaves using TRIzol (Ambion, Hillsboro, Oregon, USA) following the manufacturer’s protocol. Ribosomal RNA was depleted using a RiboZero kit (Illumina, San Diego, CA, USA) according to the manufacturer’s protocol. Next, a cDNA library was synthesized using the TruSeq Stranded Total RNA with Ribo-Zero Gold (Illumina). The cDNA library obtained was subjected to deep sequencing using the Illumina HiSeq 2500.

Raw data (raw reads) were processed using Trimmomatic^22^. Low quality reads were removed. Next, the clean reads were assembled into expressed sequence tag clusters (contigs) and de novo assembled into transcripts by using Trinity (version: 2.4) with the paired-end method^23^. The longest transcript was chosen as a unigene based on the similarity and length of a sequence for subsequent analysis. The assembled-contigs (above 150 nt) were subjected to local BLAST searches against the reference viral sequence (RefSeq) database of NCBI (the E-value cut-off was > 0.05 for local tBlastx).

### RT-PCR amplification of virus genomes

First, the partial fragment (Amplicon 2) of the rhabdovirus L gene containing a conserved polymerase motif were amplified using the previously reported degenerate primers RhabFor (GGATMTGGGGBCATCC) and RhabRev (GTCCABCCYTTTTGYC)^24^. In addition, specific primers were designed based on the contig sequences obtained above to amplify and verify the genome sequence (Table S1). Total RNA was extracted using the TRIzol Reagent (ThermoFisher Scientific) from the diseased leaves. The RT-PCR reactions were performed using the Prime ScriptTM One Step RT-PCR Kit (TaKaRa, Shiga, Japan). The amplification conditions were as follows: 50 °C for 30 min, 94 °C for 3 min, 30 cycles of 94 °C for 30 s, 48 °C for 30 s and 72 °C for 1 min, 72 °C for 10 min, 4 °C for 10 min. The resulting PCR amplicons were resolved in 1% agarose gels, stained with Ts-GelRed (TsingKe, Beijing, China), and cloned into the pMDTM19-T Vector Cloning Kit (TaKaRa). Three positive recombinant plasmids of every fragment were sequenced in both-directions.

Genomic 5′ and 3′-termini were determined using SMARTer 5′/3′ RACE kits (TaKaRa, Shiga, Japan). For the 5′-terminus, first-strand cDNA was produced with random priming according to the user manual. Rapid amplification of 5′-end was performed with the primer GPS-5′end: GATTACGCCAAGCTTGGGAAGCCCATATGTGACCCGAAGAC (identical to 13,146–13,171 of the genomic 5′-end). A band of approximately 700 bases was obtained. For the 3′-terminus, total RNA was added with a poly(A) tail using Poly(A) Polymerase (TaKaRa), then used as a template to synthesize the first-strand cDNA of the 3′-end. Rapid amplification of the 3′-end was performed with the primer GPS-3end: GATTACGCCAAGCTTTGGAGGAGAGGAGAACACATTCCCTCC (reverse complementary with 478–504 of genomic 3′-end). A band of approximately 730 bases was obtained. The PCR products of both ends were cleaned and cloned according to the manufacturer’s instructions. Eight colonies were sequenced in both directions.

### Sequence analysis

Sequences were compared with those published for rhabdoviruses using DNAMAN7.0 software. Open reading frames (ORFs) of PyRV1 were predicted using the ORF Finder program of the NCBI (https://www.ncbi.nlm.nih.gov/orffinder/). The predicted protein were verified using SMART BLAST (http://smart.embl.de/)^25^. Conserved and functional domains of the predicted proteins in PyRV1 were identified using the Conserved Domain Database (CDD) of the NCBI^26^ and the pfam database^27^. Transmembrane helices were predicted using the web based TMHMM Server v. 2.0 (https://services.healthtech.dtu.dk/service.php?TMHMM-2.0), and SignalP were used to predict signal peptide cleavage sites (https://services.healthtech.dtu.dk/service.php?SignalP-5.0). The nuclear localization and export signals were predicted by cNLS Mapper (http://nls-mapper.iab.keio.ac.jp/cgi-bin/NLS_Mapper_form.cgi)^28^ and NetNES 1.1 (https://services.healthtech.dtu.dk/service.php?NetNES-1.1)^29^, respectively. Alignment analyses of the expected amino-acid sequences of viral proteins were performed using MEGAX software, phylogenetic trees were constructed by neighbor joining algorithm with 1000 bootstrap replicates^30^.

### RT-PCR detection of PyRV1 from field samples

Based on the alignments of the nucleotide sequences encoding conserved polymerase motifs in betanucleorhabdoviruses L genes, the primers specific to PyRV1 were designed (8307F:5′-TGGAGGATATGGGGTCACCCGAT-3′, 9046R: 5′-TCAGACATGGTGATCATCGGGAAATA-3′) to detect field samples. RT-PCR was conducted using the Prime ScriptTM One Step RT-PCR Kit (TaKaRa, Shiga, Japan) according to the above-mentioned protocol. Ten plant samples with virus-like symptoms were randomly collected from plantation of *Paris polyphylla* var. *yunnanensis* in Kunming city of Yunnan province, China in July 2021.

## Results

### Symptoms on *P. polyphylla* var. yunnanensis and maintenance

**Figure 1:**
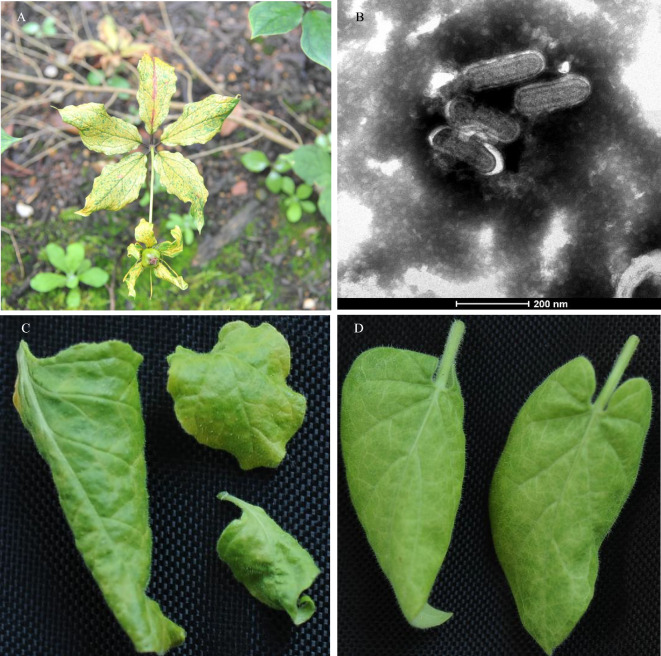
Symptoms and morphology of PyRV1. (**A**) Leaf curling, vein clearing and yellow on PyRV1 infected *Paris polyphylla* var. yannanensis. (**B**) Virus particles in the saps of PyRV1-infected *P. polyphylla* var. yannanensis plant. (**C**, **D**) Symptoms of PyRV1 on systemic leaves of *Nicotiana benthamiana* (**C**) and *N. glutinosa* (**D**) showing rolling, mottling, yellow and curling.

The *P. polyphylla* var. *yunnanensis* plants exhibited mild distortion on leaves and leaf tips rolling downward after sprouting in the spring. Vein clearing with the leaf growth and leaf distortion (Fig. 1A) and necrotic spots on leaves were observed in autumn. The infected plants failed to produce inflorescence. Approximately 5% of plants showed similar symptoms. Bacilliform-shaped virus particles of 185–220 nm length and 35–40 nm width were observed in the sap of diseased leaves of *P. polyphylla* var. *yunnanensis* (Fig. 1B). The virus was mechanically inoculated into *N. bethamiana*, *N. glutinosa*, *N. tabacum* var. K326 and *S. oleraceus*. Inoculated plants were examined twice weekly for the development of symptoms. Leaf rolling downward and mottle symptoms were observed on *N. bethamiana* and *N. glutinosa* in 6 weeks after inoculation (Fig. 1C, D). No symptoms were found on *N. tabacum* var. K326 and *S. oleraceus*. RT-PCR with the specific primers (8307F and 9046R) confirmed the presence of this virus in *N. bethamiana* and *N. glutinosa*, but absence in *N. tabacum* var. K326 and *S. oleraceus* (Figure S1A and S1B).

### Sequence analysis

To further investigate the virus infecting *P. polyphylla* var. *yunnanensis*, HTS was used to characterize the viral genomic sequence. A new plant rhabdovirus was uncovered from 2 out of 9 samples showing symptoms typical of virus infection, such as vein clearing, leave crinkle, mosaic and mottle. A total of about 25.4 M reads were generated from the HiSeq sequencing. After quality trimming and size filtering, about 24.9 M reads were used for normalization and de novo assembly. From the 15,721 assembled contigs of one sample, a contig of 13,687nucleotides showed 68.75% (37% query coverage and 4e-173 E-value) identity to Sonchus yellow net virus (SYNV) isolate DSMZ PV-0052 (MT613317), 66.24–70.82% identities (21–35% coverage and 6e-104-1e-136 E-value) to partial nucleotide sequences of other nucleorhabdovirus such as Blackcurrant-associated rhabdovirus (BCaRV) isolate Mara Eglite (OU015520), Datura yellow vein virus (DYVV) (KM823531), Zhuye pepper nucleorhabdovirus isolate ZPNu1 (ZPNRV), Cardamom vein clearing virus (CdVCV), Bacopa monnieri virus 2 (BmV2) isolate of India and Plectranthus aromaticus virus 1(PleArV1) using BLASTn. Using BLASTx, the contig shared 53.39%, 56.32% and 49.44% (45% coverage and zero E-value) identities to nucleocaspid, glycoprotein and L protein of SYNV (L32603), respectively. Five contigs of 1500–2000 bp, which also was aligned to SYNV and shared aa sequence identity from 49.0 to 53.2% with nucleocaspid, glycoprotein and L protein of SYNV, were obtained from another samples. We also detected 4 contigs of 900–3900 bp were mapped to the PMNV-cn isolate (MF509898.1) with nt sequence identity from 82.4 to 90.3%.

**Figure 2:**
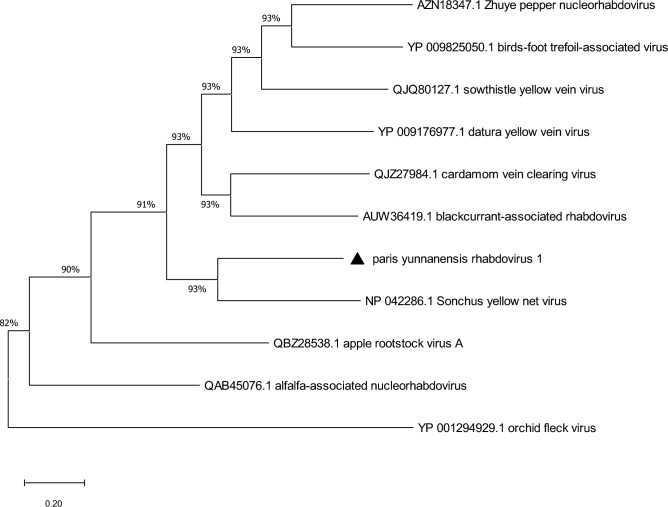
Phylogenic tree of the complete genomes of the genus *Betanucleorhabdovirus* using potato yellow dwarf virus belonging to *Alphanucleorhabdovirus* as an outgroup.

The complete genome of PyRV1 was amplified by RT-PCR (with the specific primers designed from the contigs and Sanger-sequencing was used to confirm the HTS data (Figure S1C and Figure S2). The 5′/3′-ends were determined by RACE (Figure S1D). All the RT-PCR obtained sequences, including the two ends, were assembled to compile the full-length genome sequence of 13,709 bases. The sequence was deposited in the GenBank databases under accession number OL439478. Pair wise comparison of the complete genome sequence with betanucleorhabdoviruses from GenBank showed that PyRV1 had 55.1% nt sequence identity to SYNV, 53.7% to DYVV, and 53.6% to BCaRV (Table 1). Moreover, PyRV1 was clustered within the genus *Betanucleorhabdovirus* in a clade with SYNV, DYVV and BCaRV in the phylogenetic tree based on the complete genome sequences of the selected rhabdoviruses (Fig. 2).

**Table 1:** Pairwise sequence identity (%) between PyRV1 and the other most relevant betanucleorhabdoviruses.

Virus	Acc.No	Genome (nt)	N		P		P3		M		G		L	
			nt	aa	nt	aa	nt	aa	nt	aa	nt	aa	nt	aa
SYNV	L32603	55.1	57.4	56.9	48.6	37.2	48.5	38.4	48.9	41.8	58.7	56.7	53.6	49.4
DYVV	KM823531	53.7	57.8	53.8	40.8	23.1	38.1	26.7	33.4	34.6	50.0	40.7	50.0	44.2
BCaRV	MF543022	53.6	55.9	50.6	44.1	30.5	37.4	30.9	45.8	37.5	53.3	49.0	55.5	45.7
CdVCV	MN273311	52.6	57.0	51.7	39.9	23.3	38.9	33.5	29.0	28.2	52.7	45.3	51.8	45.5
SYVV	MT185675	51.9	56.7	52.4	40.3	23.9	32.7	24.4	36.3	35.1	48.9	44.3	50.0	45.1
ZPNRV	MH323437	41.3	42.8	55.7	39.5	23.6	32.3	26.1	36.3	34.1	33.9	44.4	53.0	44.9

### Genome organization of PyRV1

**Figure 3:**
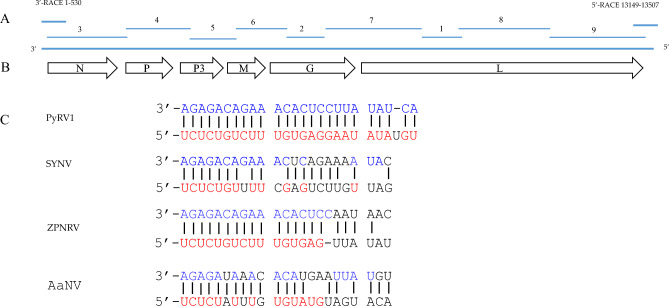
Analysis of PyRV1 genome. (**A**) RT-PCR amplification for the complete genome of PyRV1. (**B**) Genome organization of PyRV1. (**C**) Complementary structure between the 3′ and 5′ termini in the genome of PyRV1 and comparison with SYNV, ZPNRV and AaNV. Vertical lines indicate nucleotides that are complementary between the leader and trailer sequences. Red and blue marked nucleotides are identical to PyRV1. *AaNV* alfalfa associated nucleorhabdovirus, *SYNV* sonchus yellow net nucleorhabdovirus, *ZPNRV* Zhuye pepper nucleorhabdovirus.

The genome of PyRV1 had an organization similar to SYNV. The genome contains 6 ORFs in the order 3′–N–P–P3–M–G–L–5′, which were similar to most nucleorhabdoviruses. The PyRV1 genome included a 201-nt 3′-leader and a 162-nt 5′-trailer. The 3′-leader and the 5′-trailer of PyRV1sharing 23 complete complementary nucleotides that could potentially form a panhandle structure common to all known rhabdovirus genomes. The 3′-leader of PyRV1 had 17 nt and 12 nt identities to those of ZPNRV and SYNV, respectively (Fig. 3). The PyRV1 ORFs were separated by highly conserved intergenic region, which were composed of a polyadenylation signal of the preceding gene, non-transcribed intergenic spacer and transcriptional start of the following gene (Table 2). The PyRV1 consensus intergenic region (IGR) sequence 3′-UAUAUUCUUUUU GG UUG-5′ was identical to that of SYNV except for the L/5′-trailer. There was no transcriptional start site UUG in the L/5′-trailer.

**Table 2:** Pairwise sequence identity (%) between PyRV1 and the other most relevant betanucleorhabdoviruses.

Gene junction	Polyadenylation	Intergenic spacer	Transcription start
3-Leader/N	^126^UAAUUUCUUUUU^137^	^138^GG^139^	^140^UUG^142^
N/P	^1698^UAUAUUCUUUUU^1709^	^1710^GG^1711^	^1712^UUG^1714^
P/P3	^2905^UAUAUUCUUUUU^2916^	^2917^GG^2918^	^2919^UUG^2921^
P3/M	^4077^UAUAUUCUUUUU^4088^	^4089^GG^4090^	^4091^UUG^4093^
M/G	^5132^UAUAUUCUUUUU^5143^	^5144^GG^5145^	^5146^UUG^5148^
G/L	^7168^UAUAUUCUUUUU^7179^	^7180^GG^7181^	^7182^UUA^7184^
L/5-trailer	^13554^UAUAUUCUUUUU^13565^	^13566^GGCC^13569^	
SYNVconsensus	UAUAUUCUUUUU	GG	UUG

### Predicted protein features of PyRV1

The features of PyRV1-encoding proteins are listed in Tables 1 and 3. The N gene (ORF1) was 1419 nt in length (202-1620) which encodes the 52.9 kDa nucleocapsid protein. Sequence comparison analysis showed that the N protein of PyRV1 shared 50.6–56.9% aa identity with, ZPNRV, DYVV, Sowthistle yellow vein virus (SYVV), CdVCV, and BCaRV, which are members of the genus *Betanucleorhabdovirus* (Table 1). The N protein contains a cytoplasmic and nuclear localization signal (NLS) (cNLS) at aa 484–494, and a leucine-rich nuclear export signal (NES) was predicted at positions 39 (Table 3).The P gene (ORF2) was 1023 nt (1821–2843) and encodes a phosphoprotein of 37.4 kDa with a nuclear localization signal at aa 140–174 and two NESs predicted at positions 280 and 284. The P protein shared 23.1–37.2% aa identities with SYNV, BCaRV, SYVV, ZPNRV, CdVCV and DYVV. The P3 gene (ORF3) was 966 nt in length (2980–3945) and encodes a putative 35.3 kDa movement protein. The P3 protein was predicted to contain a partial cytoplasmic and three NLSs. The P3 shared 24.4–38.4% aa identities with SYNV, CdVCV, BCaRV, DYVV, ZPNRV and SYVV. The M gene (ORF4) was 855 nt (4149–5003) and encoded a matrix protein of 31.2 kDa with two predicted NLSs at positions 229–243, no NES was detected. The M shared 28.2–41.8% aa identity with SYNV, BCaRV, SYVV, DYVV, ZPNRV and CdVCV. The G gene was 1899 nt long (5170–7068) and encodes a 69.5 kDa glycoprotein and contained one predicted NLS and one NES at L13. Glycoprotein shared 40.7–56.7% aa identity with SYNV, BCaRV, CdVCV, ZPNRV, SYVV and DYVV. The L gene was 6321nt in length (7221–13,547) in size and encodes a 231.9 kDa polymerase protein. The aa sequence of L protein contained a nuclear localization signal at position 1644–1667. Three NESs were predicted at positions L 240, 2027 and I2026. L protein shared 44.2–49.4% aa identity with SYNV, BCaRV, CdVCV, SYVV, ZPNRV and DYVV (Table 1). In addition, only the G protein contained a predicted transmembrane domain at aa 552–574 (GLFGGIAKVFILIICCIIVYI) and a signal peptide site at aa1-25 (e = 0.7273). The NLS and NES predictions for each ORF aa sequence indicate that PyRV1 may replicate in the nucleus of infected cells.

**Table 3:** Analysis of PyRV1 sequence for nuclear localization signals (NLS), nuclear export signals, transmembrane domains and Signal peptite.

ORF	Protein	Partite	Predicted NLS (cNLS Mapper score)	Predicted nuclear export signal site
1	N	Monopartite	^484^PSRKRMFQEAI (4.5)	L^39^
2	P	Bipartite	^140^RKVVKAARKDALQRNTKGIKQPESKEIEQEKTPKT (5.6)	I^280,284^
3	P3	Bipartite	EESYNKSSDCVVFHSAPITGSCGEIRVKKQDMT (3.5)	L^87,92^, D^94^
4	M	Monopartite	^229^RPKIMKRKKIMRQLF (7)	Not detected
5	G	Bipartite	^155^KTIPKGGVHYITNSGYAECEYFSDNTKCARDY (4.6)	L^13^
6	L	Bipartite	^1644^DQATLKRKGTLACMMDRAKRARLI (11.5)	L^240,2027^, I^2026^

### Conserved residues and motifs

The amino acid sequences of the predicted PyRV1 proteins were aligned with those of available plant rhabdoviruses. The alignment of conserved residues and motifs revealed that PyRV1 shared some conserved residues and motifs in N, G and L proteins with other nuleorhabdoviruses. No conserved residues were identified in P, P3 and M between PyRV1 and other plant rhabdoviruses. Thirteen conserved positions and one motif (364–366,WKY) were identified in the N protein, 10 conserved cysteine residues (79, 83, 242, 252, 263, 339, 343, 380, 395 and 427) and 2 tryptophan residues (position 231 and 405) in G protein. Sixty four conserved amino acid residues were identified in L protein. Several conserved domains involved in the RNA dependent RNA polymerase activity function^31,32^ such as the RxWGHP motif, Pre-motif A (GxxxKERE), Motif A (DExKWNxxxE), Motif B (GxEGxRQKxWT), Motif C (GxGDNQ) and Motif D (GLPxKxxExWxSx_7_Kx_13_K) were located at position 364–369, 560–568, 639–644, 711–721and 743–748, respectively. The underlined residues in motifs were specific to mononegaviruses^31,33,34^.

### Phylogenetic analysis of the complete genome and predicted proteins

To understand phylogenetic relationship between PyRV1 and other plant rhabdoviruses, the maximum likelihood phylogenic trees were generated using MEGA X program. The phylogenetic tree based on complete betanucleorhabdoviral genome inferred that PyRV1 is most closely related to SYNV, with at high bootstrap value > 88 (Fig. 2). The phylogenetic tree of the deduced aa sequences of L proteins of the *Betarhabdovirinae* sub-family also indicated the closest relative of PyRV1 was SYNV. These viruses formed a cluster clearly separated from other betanucleorhabdoviruses, alphanucleorhabdoviruses, gamanucleorhabdovirus, cytorhabdoviruses, dichorhaviruses and varicosaviruses formed separate clusters well separated from each other (Figure S3) as would be expected. Phylogenetic relationships for all of the other five proteins also showed that PyRV1 clustered together with SYNV, CdVCV, BCaRV, DYVV and SYVV of the genus *Betanucleorhabdovirus* (not shown).

### RT-PCR detection for field samples

Ten field samples, collected from the plantation of *P. polyphylla* var. *yunnanensis* were detected by using PyRV1 specific primer derived from L conversed motifs. A 700-bp specific PCR product from one sample was amplified (Figure S4). The amplicon was sequenced to verify the infection of PyRV1 to this sample (data not shown).

## Discussion

The presence of a novel nucleorhabdovirus in *P. polyphylla* var. *yunnanensis* was established by using EM and HTS technologies. The morphological studies by TEM confirmed that the etiologic agent was associated with a rhabdovirus. The morphological results were verified by HTS and targeted amplicon sequencing. The genome of PyRV1 was organized similarly to those of SYNV, SYVV, DYVV, BCaRV and bird’s-foot trefoil-associated virus 1 (BFTV-1). The genome contained 6 genes in the order 3′–N–P–P3–M–G–L–5′, each gene being separated by a conserved IGR (UUCUUUUU GG UUG), that was common to SYNV (Table 2). The genome nucleotide sequence was observed to share approximately 41.3% to 55.1% identities (Table 1) between PyRV1 and other betanucleorhabdoviruses. Sequence identities of all ORFs provided evidence that PyRV1was the most similar to the respective counterparts in SYNV, DVYY, SYVV, BCaRV, ZPNRV and CdVCV of the genus *Betanucleorhabdovirus*. Phylogenetic analysis based on the complete genome, and aa sequences of the L and N proteins showed PyRV1 clustered within the branch of betanucleorhabdoviruses including SYNV, BCaRV, DYVV, SYVV, CdVCV (Fig. 2 and S3). Nucleorhabdoviruses (genera *Alphanucleorhabdovirus*, *Betanucleorhabdovirus*, *Gammanucleorhabdovirus*) replicate in viroplasms in the host cell nucleus^13,35^. As has been reported for both SYNV and DYVV^36,37^, the NLS and NES were also observed in the encoded proteins of PyRV1 (Table 3). The PyRV1 P3 protein, a putative movement protein contained a predicted NLS, but no NES^38–40^. The presence of conserved residues and motifs in the N, G, and L proteins, especially the canonical GHP motif Pre-motif A, motif A, B, C, and D in the L protein suggested that they have similar respective functions and/or structural features among plant rhabdoviruses^32,41^. Consequently, complete genome alignments among PyRV1 and other available plant rhabdoviruses showed extremely divergent of these nt and aa sequences, as it is commonly observed for different plant rhabdoviruses^13,31^. Based on the molecular aspects, especially the highest nucleotide sequence identity of 55.1% between PyRV1 and other plant rhabdoviruses is lower than the identity threshold level (75%) for establishing a new species of the genus *Betanucleorhabdovirus*. Therefore, PyRV1 should be considered as a new species in the genus *Betanucleorhabdovirus*.

In addition, a new species assigned to the genus *Betanucleorhabdovirus* has other two characteristics: should occupy different ecological niches (differences in hosts and/or arthropod vectors), can be clearly distinguished in serological tests or by nucleic acid hybridization^15^. Attempts were taken to isolate the virus from single local lesion through inoculation into *Chenopodium quinoa* leaves but could not possible. So, the virus was directly inoculated into *N. benthamiana* and *N. glutinosa*. The inoculated *N. benthamiana* and *N. glutinosa* plants exhibited vein clearing symptom that was similar to the symptoms on *P. polyphylla* var. *yunnanensis*. Inoculation followed by verification of amplicon sequencing. The virus was mechanically smeared with the pestle onto 1-year seedlings of *P. polyphylla* var. *yunnanensis*. The inoculated plants wilted after 1 week. It was not possible to detect virus from the withered leaves. When the virus was inoculated into more than 1-year aged *P. polyphylla* var. *yunnanensis* plants, did not exhibit the virial symptoms and could not detect the virus. In nature, plant rhabdoviruses are transmitted by insect vectors such aphids, leafhoppers or planthoppers^21^. SYNV is transmitted by aphid. So, PyRV1 may also be transmitted by aphid. However, we could not investigate the aphid in the diseased field. So, we are not sure about the vector. As a wild plant, *P. polyphylla* var*. yunnanensis* has been cultivated in a large scale for more than 20 years, several viruses have been detected in *P. polyphylla* var. *yunnanensis*^7–12^. However, back inoculation into *P. polyphylla* var. *yunnanensis* is a dilemma. The leaves of inoculated *P. polyphylla* var. *yunnanensis* exhibited clear vein and yellow symptoms similar to naturally infection (Fig. S5), and a weak targeted fragment also was amplified from inoculated leaves of *P. polyphylla* var. *yunnanensis* inoculated by PyRV1 (Fig. S6), however, no particle was observed in the saps of the inoculated leaves. Medicinal plant hosts like *Paris* sp., *Panax* sp. and *Polygonutum* sp. plants may pose additional problems in fulfilling the Koch’s postulate by smearing inoculation because the abundant secondary metabolites such as saponins and polysaccharides, which may possess the potential antiviral activities present in the leaf mesophyll, interfere with virus infection^3,42,43^.

In the present study, a novel betanucleorhabdovirus (PyRV1) causing vein clearing and leaf crinkle disease was discovered in *P. polyphylla* var. *yunnanensis*, and was characterized based on morphological and molecular aspects. This virus was transferred to *N. bethamiana* and *N. glutinosa* by mechanical smearing inoculation. However, back inoculation to *P. polyphylla* var. *yunnanensis* by insect vector is needed to fulfill the Koch’s postulate. Thus, further research is needed to identify natural vectors of this virus as well as alternative hosts, develop a serological assay technique and fluorescent viral protein localization to provide strong evidences of species demarcation.

## Conclusions

This study identified a novel negative-sense ssRNA virus of paris yunnanensis nucleorhabdovirus (PyRV1), which we suggest belonged to a new species in the genus *Betanucleorhabdovirus*, based on the study of morphology and analysis of genomic organization, sequence similarity, and phylogeny. Our results also revealed the significant diversities between PyRV1 and other nucleorhabdoviruses in terms of gene sequences. Further study was needed to characterize this virus in terms of host range, morphogenesis and its insect vectors.

## Supplementary Information

Supplementary Information.

## Data Availability

All the data presented in this study are available in this article and Supplementary Materials.
