# Supplementary material for: Identification, molecular characterization and phylogenetic analysis of a novel nucleorhabdovirus infecting Paris polyphylla var. yunnanensis
Source: Sci Rep. 2023 Jun 20;13:10040. doi: 10.1038/s41598-023-37022-2 (PMC10282024; doi:10.1038/s41598-023-37022-2)
Supplement: Supplementary file 1 — Supplementary Information. [file 41598_2023_37022_MOESM1_ESM.pptx]

## Slide 1
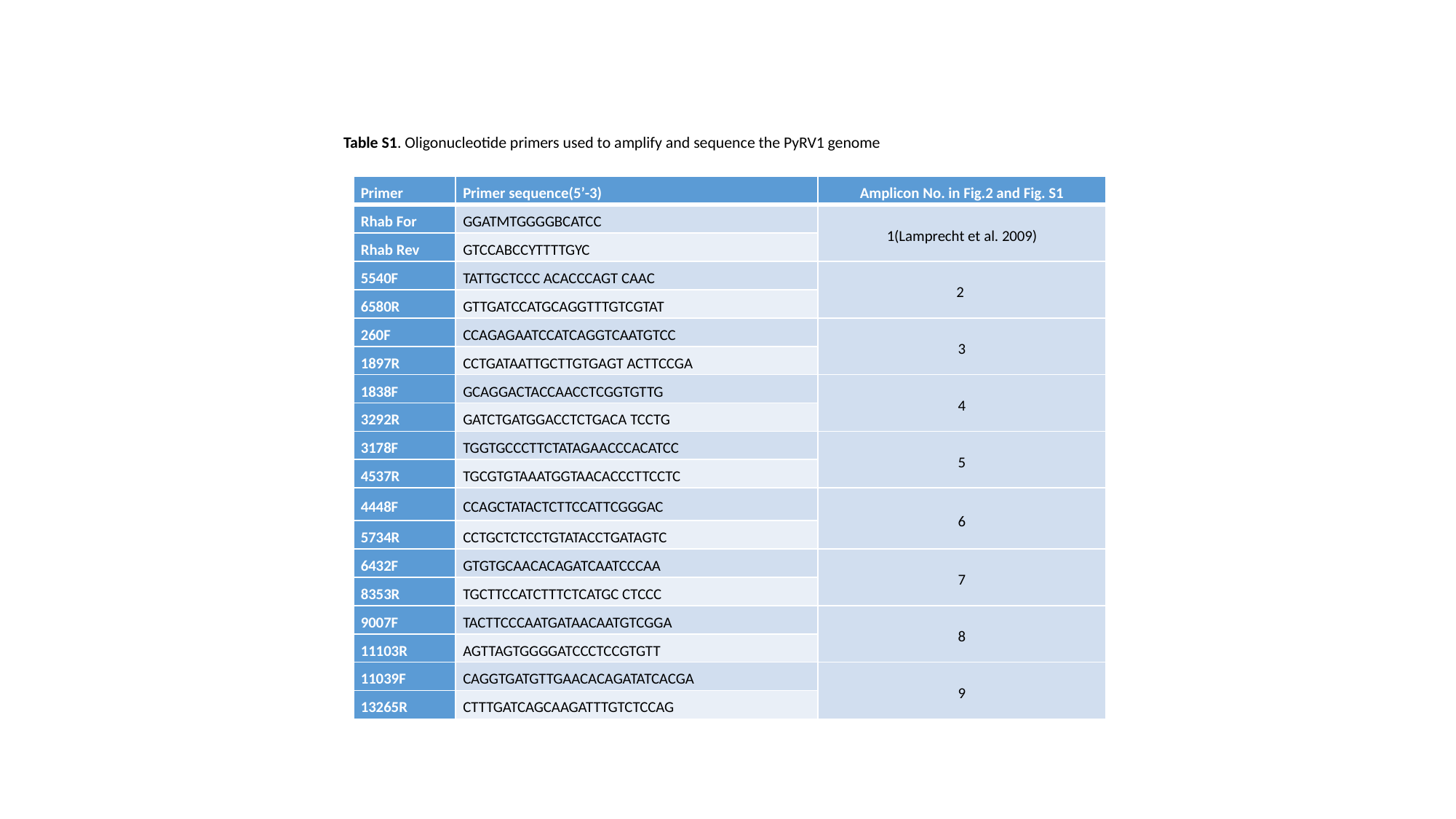

Table S1. Oligonucleotide primers used to amplify and sequence the PyRV1 genome
| Primer | Primer sequence(5’-3) | Amplicon No. in Fig.2 and Fig. S1 |
| --- | --- | --- |
| Rhab For | GGATMTGGGGBCATCC | 1(Lamprecht et al. 2009) |
| Rhab Rev | GTCCABCCYTTTTGYC | |
| 5540F | TATTGCTCCC ACACCCAGT CAAC | 2 |
| 6580R | GTTGATCCATGCAGGTTTGTCGTAT | |
| 260F | CCAGAGAATCCATCAGGTCAATGTCC | 3 |
| 1897R | CCTGATAATTGCTTGTGAGT ACTTCCGA | |
| 1838F | GCAGGACTACCAACCTCGGTGTTG | 4 |
| 3292R | GATCTGATGGACCTCTGACA TCCTG | |
| 3178F | TGGTGCCCTTCTATAGAACCCACATCC | 5 |
| 4537R | TGCGTGTAAATGGTAACACCCTTCCTC | |
| 4448F | CCAGCTATACTCTTCCATTCGGGAC | 6 |
| 5734R | CCTGCTCTCCTGTATACCTGATAGTC | |
| 6432F | GTGTGCAACACAGATCAATCCCAA | 7 |
| 8353R | TGCTTCCATCTTTCTCATGC CTCCC | |
| 9007F | TACTTCCCAATGATAACAATGTCGGA | 8 |
| 11103R | AGTTAGTGGGGATCCCTCCGTGTT | |
| 11039F | CAGGTGATGTTGAACACAGATATCACGA | 9 |
| 13265R | CTTTGATCAGCAAGATTTGTCTCCAG | |

## Slide 2
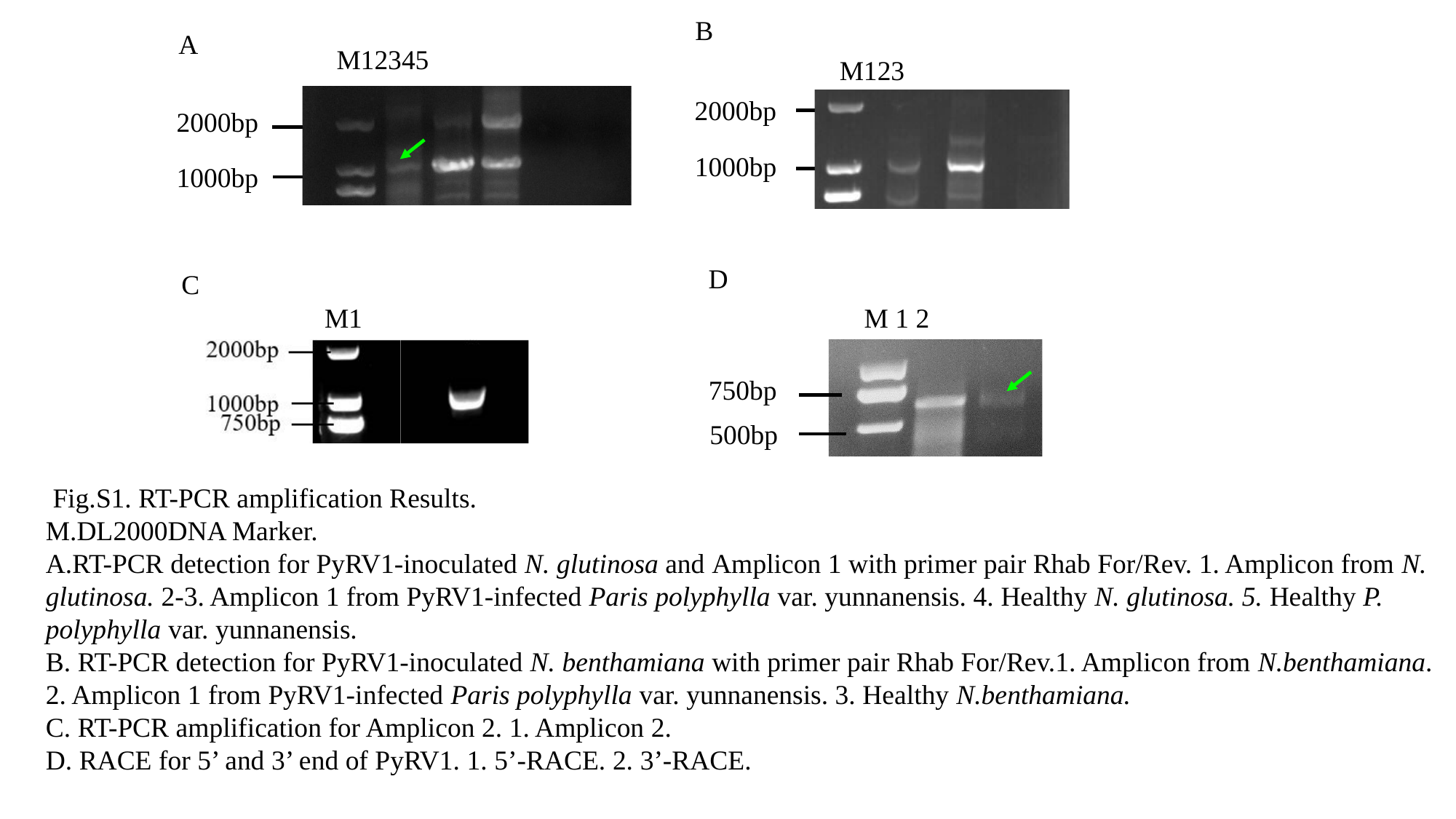

B
A
M12345
M123
2000bp
2000bp
1000bp
1000bp
D
C
M1
M 1 2
750bp
500bp
 Fig.S1. RT-PCR amplification Results.
M.DL2000DNA Marker.
A.RT-PCR detection for PyRV1-inoculated N. glutinosa and Amplicon 1 with primer pair Rhab For/Rev. 1. Amplicon from N. glutinosa. 2-3. Amplicon 1 from PyRV1-infected Paris polyphylla var. yunnanensis. 4. Healthy N. glutinosa. 5. Healthy P. polyphylla var. yunnanensis.
B. RT-PCR detection for PyRV1-inoculated N. benthamiana with primer pair Rhab For/Rev.1. Amplicon from N.benthamiana. 2. Amplicon 1 from PyRV1-infected Paris polyphylla var. yunnanensis. 3. Healthy N.benthamiana.
C. RT-PCR amplification for Amplicon 2. 1. Amplicon 2.
D. RACE for 5’ and 3’ end of PyRV1. 1. 5’-RACE. 2. 3’-RACE.

## Slide 3
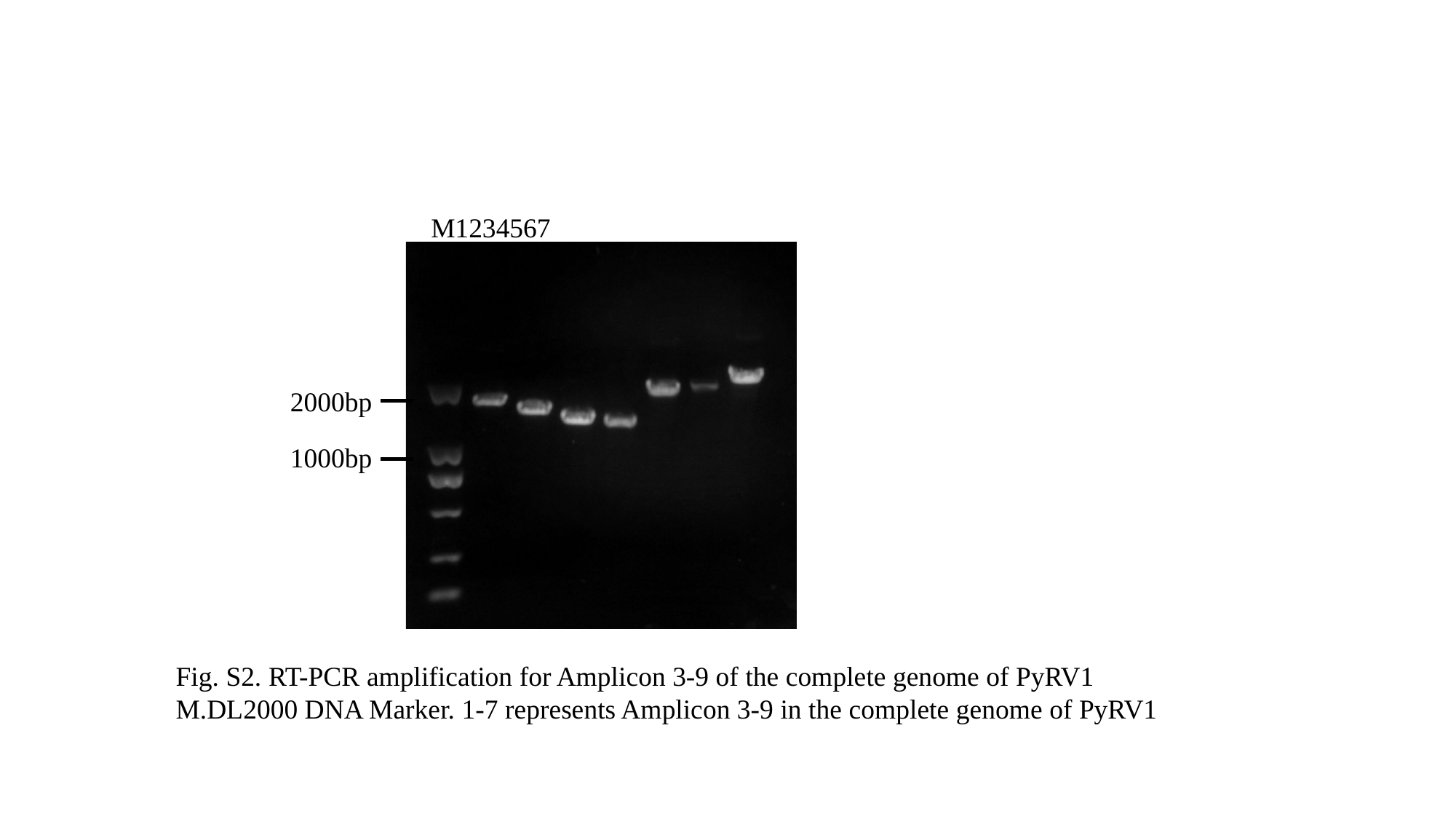

M1234567
2000bp
1000bp
Fig. S2. RT-PCR amplification for Amplicon 3-9 of the complete genome of PyRV1
M.DL2000 DNA Marker. 1-7 represents Amplicon 3-9 in the complete genome of PyRV1

## Slide 4
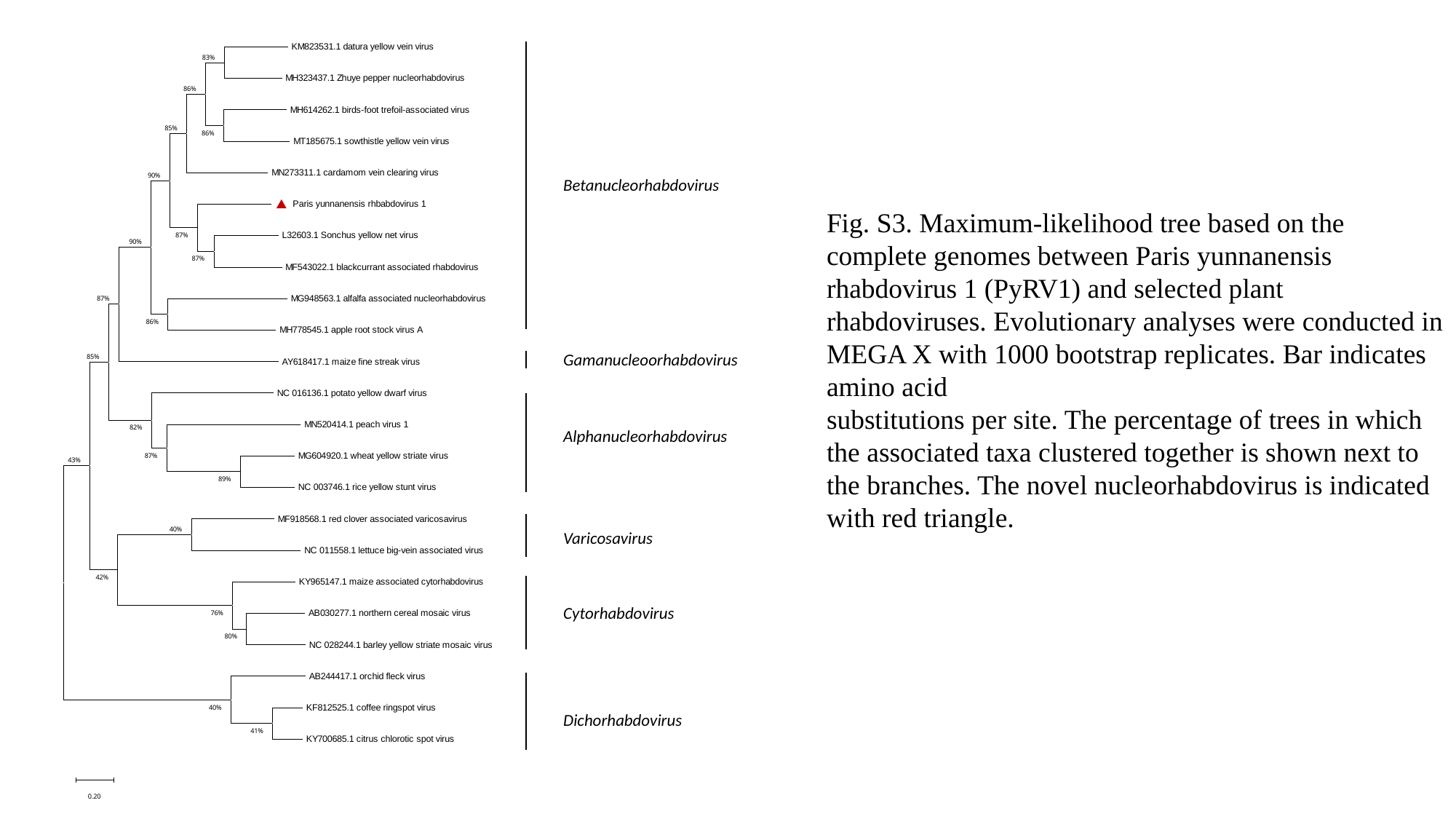

Betanucleorhabdovirus
Fig. S3. Maximum-likelihood tree based on the complete genomes between Paris yunnanensis rhabdovirus 1 (PyRV1) and selected plant rhabdoviruses. Evolutionary analyses were conducted in MEGA X with 1000 bootstrap replicates. Bar indicates amino acid
substitutions per site. The percentage of trees in which the associated taxa clustered together is shown next to the branches. The novel nucleorhabdovirus is indicated with red triangle.
Gamanucleoorhabdovirus
Alphanucleorhabdovirus
Varicosavirus
Cytorhabdovirus
Dichorhabdovirus

## Slide 5
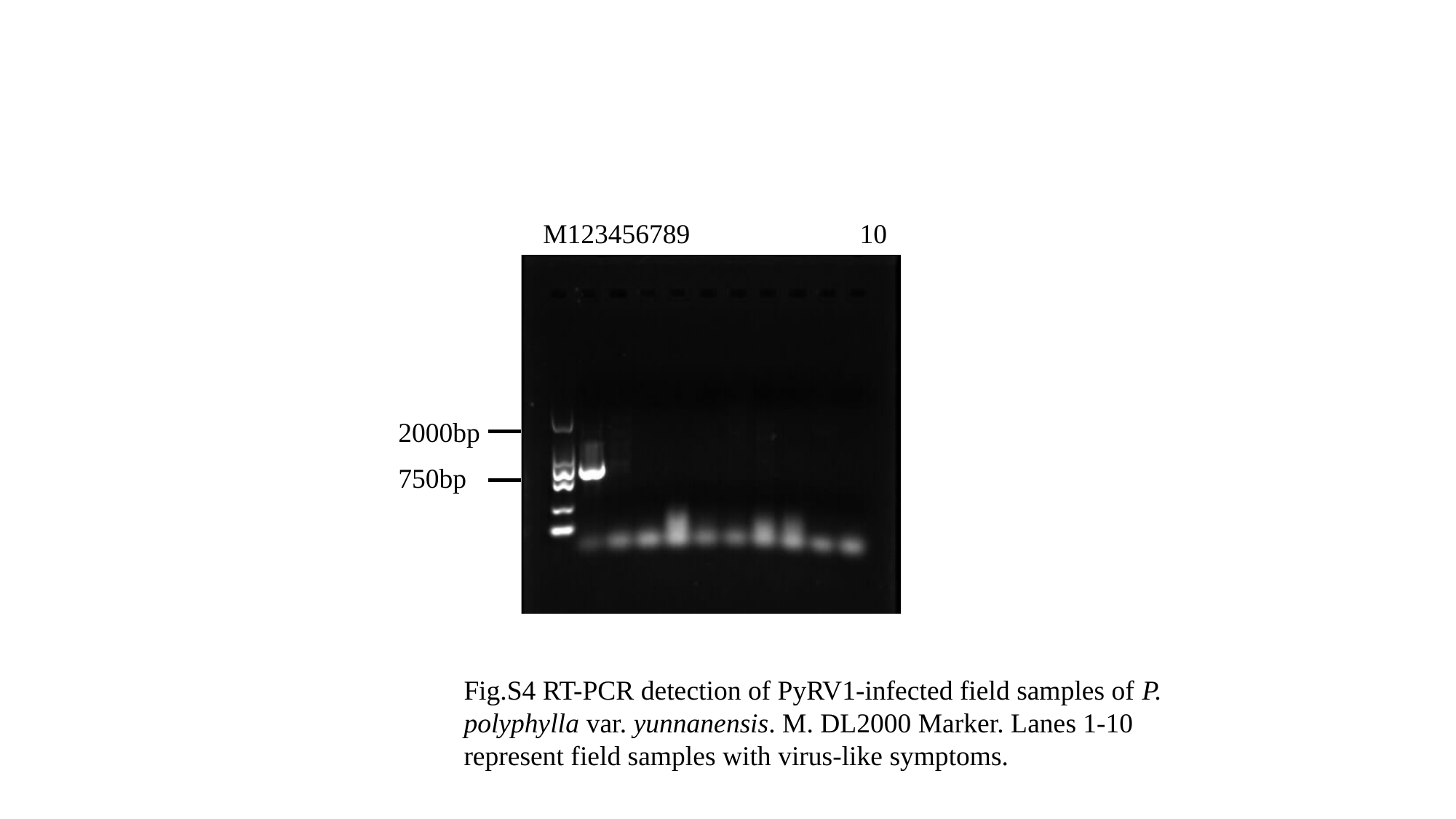

M123456789
10
2000bp
750bp
Fig.S4 RT-PCR detection of PyRV1-infected field samples of P. polyphylla var. yunnanensis. M. DL2000 Marker. Lanes 1-10 represent field samples with virus-like symptoms.

## Slide 6
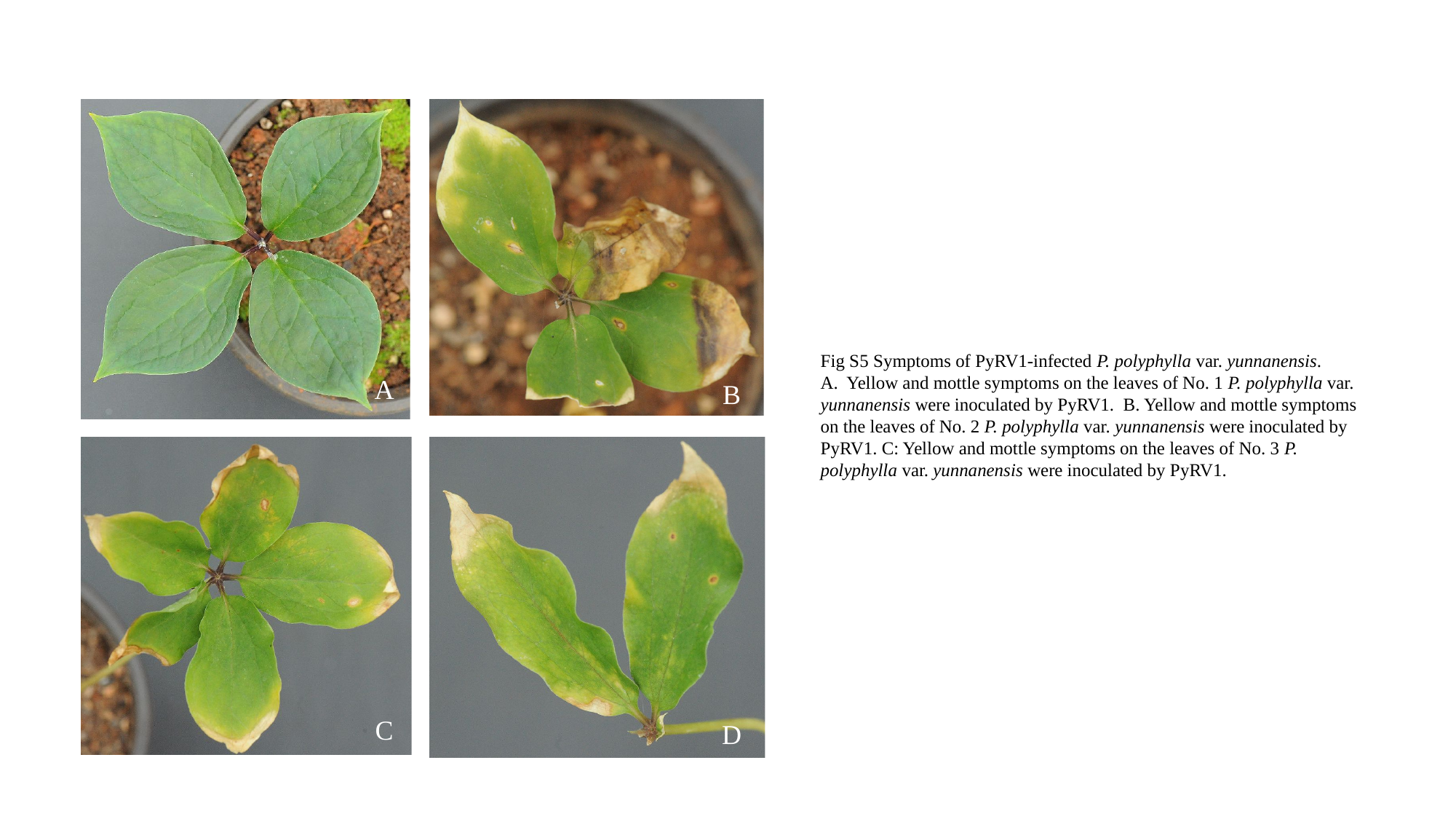

A
B
C
D
Fig S5 Symptoms of PyRV1-infected P. polyphylla var. yunnanensis.
A. Yellow and mottle symptoms on the leaves of No. 1 P. polyphylla var. yunnanensis were inoculated by PyRV1. B. Yellow and mottle symptoms on the leaves of No. 2 P. polyphylla var. yunnanensis were inoculated by PyRV1. C: Yellow and mottle symptoms on the leaves of No. 3 P. polyphylla var. yunnanensis were inoculated by PyRV1.

## Slide 7
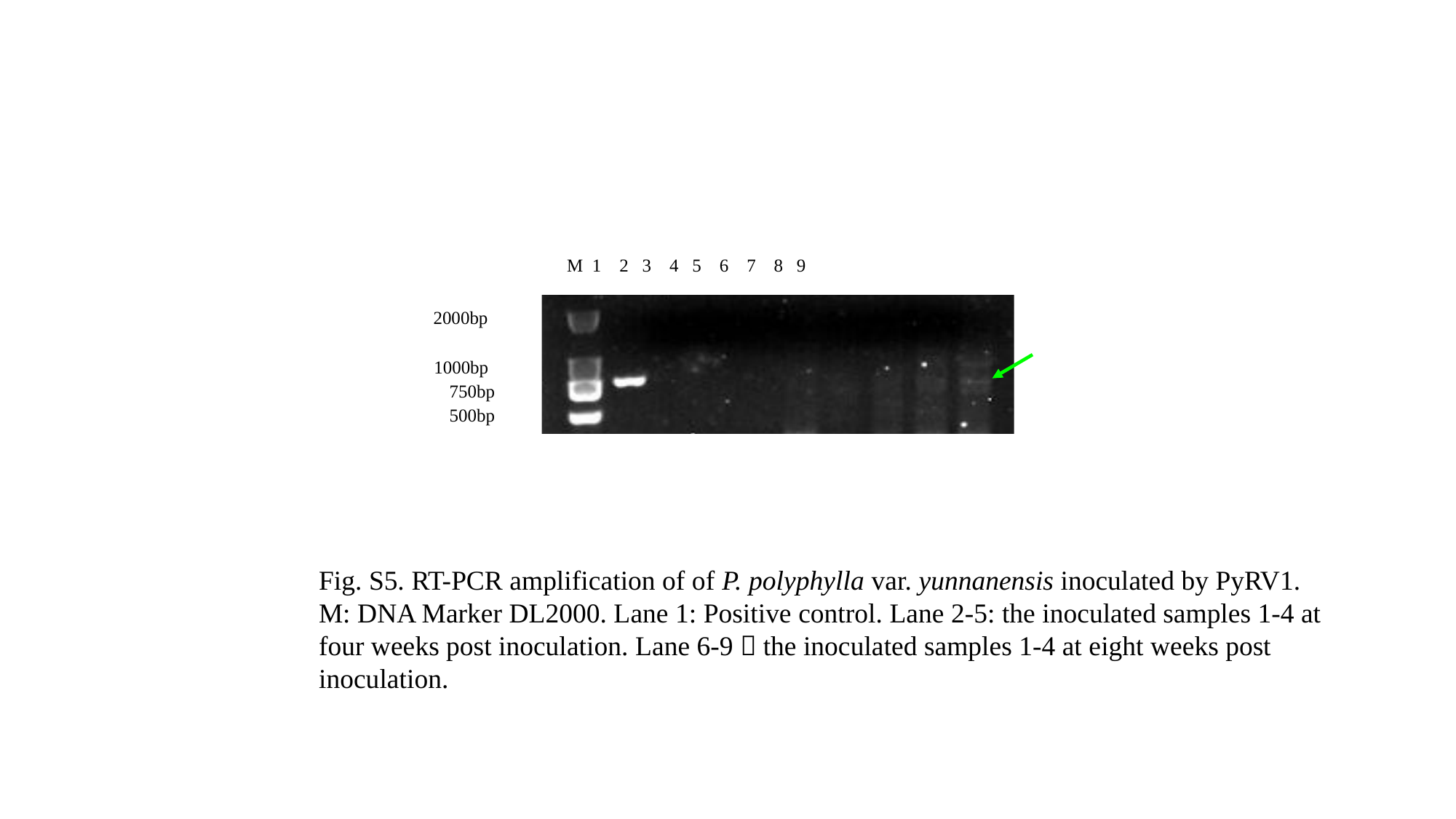

M 1 2 3 4 5 6 7 8 9
2000bp
1000bp
750bp
500bp
Fig. S5. RT-PCR amplification of of P. polyphylla var. yunnanensis inoculated by PyRV1. M: DNA Marker DL2000. Lane 1: Positive control. Lane 2-5: the inoculated samples 1-4 at four weeks post inoculation. Lane 6-9：the inoculated samples 1-4 at eight weeks post inoculation.
